# Intrathecal lidocaine pretreatment attenuates immediate neuropathic pain by modulating Nav_1.3 _expression and decreasing spinal microglial activation

**DOI:** 10.1186/1471-2377-11-71

**Published:** 2011-06-16

**Authors:** Kuang-I Cheng, Chung-Sheng Lai, Fu-Yuan Wang, Hung-Chen Wang, Lin-Li Chang, Shung-Tai Ho, Hung-Pei Tsai, Aij-Li Kwan

**Affiliations:** 1 Graduate Institute of Medicine, College of Medicine, Kaohsiung Medical University, Kaohsiung, Taiwan; 2 Department of Anesthesiology, Kaohsiung Medical University Hospital, Kaohsiung, Taiwan; 3 Department of Anesthesiology, Faculty of Medicine, College of Medicine, Kaohsiung Medical University, Kaohsiung, Taiwan; 4 Department of Surgery, Faculty of Medicine, College of Medicine, Kaohsiung Medical University, Kaohsiung, Taiwan; 5 Division of Plastic and Reconstructive Surgery, Department of Surgery, Kaohsiung Medical University Hospital, Kaohsiung, Taiwan; 6 Department of Neurosurgery, Chang Gung Memorial Hospital-Kaohsiung Medical Center, Chang Gung University College of Medicine, Kaohsiung, Taiwan; 7 Department of Microbiology, Faculty of Medicine, College of Medicine, Kaohsiung Medical University, Kaohsiung, Taiwan; 8 Department of Anesthesiology, Tri-Service General Hospital National Defense Medical Center, Taipei, Taiwan; 9 Department of Neurosurgery, Kaohsiung Medical University, Kaohsiung, Taiwan

## Abstract

**Background:**

Intrathecal lidocaine reverses tactile allodynia after nerve injury, but whether neuropathic pain is attenuated by intrathecal lidocaine pretreatment is uncertain.

**Methods:**

Sixty six adult male Sprague-Dawley rats were divided into three treatment groups: (1) sham (Group S), which underwent removal of the L_6 _transverse process; (2) ligated (Group L), which underwent left L_5 _spinal nerve ligation (SNL); and (3) pretreated (Group P), which underwent L_5 _SNL and was pretreated with intrathecal 2% lidocaine (50 μl). Neuropathic pain was assessed based on behavioral responses to thermal and mechanical stimuli. Expression of sodium channels (Nav_1.3 _and Nav_1.8_) in injured dorsal root ganglia and microglial proliferation/activation in the spinal cord were measured on post-operative days 3 (POD_3_) and 7 (POD_7_).

**Results:**

Group L presented abnormal behavioral responses indicative of mechanical allodynia and thermal hyperalgesia, exhibited up-regulation of Nav_1.3 _and down-regulation of Nav_1.8_, and showed increased microglial activation. Compared with ligation only, pretreatment with intrathecal lidocaine before nerve injury (Group P), as measured on POD_3_, palliated both mechanical allodynia (*p *< 0.01) and thermal hyperalgesia (*p *< 0.001), attenuated Nav_1.3 _up-regulation (*p *= 0.003), and mitigated spinal microglial activation (*p *= 0.026) by inhibiting phosphorylation (activation) of p38 MAP kinase (*p *= 0.034). p38 activation was also suppressed on POD_7 _(*p *= 0.002).

**Conclusions:**

Intrathecal lidocaine prior to SNL blunts the response to noxious stimuli by attenuating Nav_1.3 _up-regulation and suppressing activation of spinal microglia. Although its effects are limited to 3 days, intrathecal lidocaine pretreatment can alleviate acute SNL-induced neuropathic pain.

## Background

Peripheral nerve injury caused by disease or surgery, such as limb and colon amputation, nerve dissection, mastectomy, and thoracotomy, often results in neuropathic pain [1]. It is a neglected problem for which very few patients are referred for surgical management [2]. Patients who develop postsurgical chronic neuropathic pain suffer from acute postoperative pain and have intraoperative nerve damage [3]. Nearly 50% of patients with neuropathic pain receive inadequate pain relief, and conventional analgesics are generally ineffective [4]. Systemic or regional analgesic treatment before tissue damage occurs has proven effective for treating postoperative pain [5-7]. Pre-incisional intraspinal administration of local anesthetics inhibits nerve impulses in response to noxious stimuli and reduces postoperative morphine requirements [8]. Although treating injured peripheral nerves with local anesthetic can inhibit neuropathic pain [9,10], the effects of intrathecal lidocaine pretreatment on neuropathic pain have not been established. Because the intensity of acute postoperative pain correlates with the risk of developing a persistent pain state [3], early therapy of acute postoperative pain is important.

Lidocaine, the local anesthetic most commonly used in clinical practice, confers anti-arrhythmic and nerve conduction-blocking effects by acting on voltage-gated sodium channels. Intravenous administration of lidocaine also alleviates the tactile allodynia associated with neuropathic pain [11]. Intrathecal lidocaine after peripheral nerve injury reverses well-developed tactile allodynia either through interaction with eicosanoid systems in the spinal dorsal horn or by attenuating phosphorylation of p38 mitogen-activated protein (MAP) kinase in activated microglia of the spinal cord [12-14]. However, the use of intrathecal lidocaine pretreatment to attenuate neuropathic pain is not a recognized therapeutic option. A recent study in rodents found that intrathecal lidocaine administration before injection of formalin into the hindpaw suppresses nociceptive behavior and Fos expression by diminishing central hyperexcitability [15]. Moreover, pretreatment with intrathecal injection of 4% lidocaine delays the onset of neuropathic pain [16]. Nonetheless, pretreatment intrathecal lidocaine does not reduce the incidence of autotomy after axotomy [17]. Although intrathecal low-dose lidocaine (100 μg in 10 μl) induces a transient, flaccid leg paralysis, it does not prevent tactile hypersensitivity [18]. To elucidate the potential of intrathecal lidocaine pretreatment for the alleviation of postoperative neuropathic pain, we administered intrathecal 2% lidocaine to mimic the clinical use of regional anesthesia before surgery. The effectiveness of intrathecal lidocaine pretreatment in reducing neuropathic pain was evaluated by behavioral testing, by measuring dysregulation of sodium channel α-subunit (Nav_1.3 _and Nav_1.8_) expression in dorsal root ganglia (DRGs), and by measuring spinal microglial activation in the spinal cord. We found that intrathecal lidocaine pretreatment alleviated pain-related thermal hyperalgesia and mechanical allodynia, attenuated up-regulation of Nav_1.3 _in the injured DRG, and significantly reduced spinal microglial activation on post-operative day 3 (POD_3_). The levels of phosphorylated (activated) p38 MAP kinase (p-p38) were also diminished on POD_3 _and POD_7_. Therefore, intrathecal lidocaine administration before peripheral nerve injury can attenuate neuropathic pain for 3 days.

## Methods

### Experimental Animals and Groups

Sixty six adult male Sprague-Dawley rats weighting 300-350 g were used in this study. The animals were housed in plastic cages with soft bedding under a 12 h light-dark cycle (light cycle from 6 am to 6 pm; dark cycle from 6 pm to 6 am) with free access to food and water. The rats were kept for at least 7 days under these conditions before surgery. All experimental procedures were approved by the Kaohsiung Institutional Animal Care and Use committee. The rats were divided into three experimental groups: (1) Sham (Group S, n = 22); animals in this group had their left sixth lumbar spine transverse process surgically removed and received local lidocaine on subcutaneous tissues and paraspinal muscles, but were not subjected to spinal nerve ligation (SNL) and received no intrathecal lidocaine. (2) Ligated (Group L, n = 22); animals in this group were subjected to surgery with SNL and received local lidocaine on subcutaneous tissues and paraspinal muscles, but were not administered intrathecal lidocaine. (3) Lidocaine Pretreated (Group P, n = 22); animals in this group were subjected to surgery with SNL and received local lidocaine on subcutaneous tissues and paraspinal muscles, and were also pretreated with intrathecal 2% lidocaine. The animals were kept under the same conditions throughout the study. All experimental rats underwent thermal and mechanical pain tests (allodynia) 1 day before surgery (pre-OP), and 3, 5, 7, 14, and 28 days postoperatively (POD_3_, POD_5_, POD_7_, POD_14_, and POD_28_, respectively).

### Intrathecal Lidocaine Pretreatment

Before intrathecal lidocaine pretreatment, each rat was handled by the operator to minimize stress-induced analgesia. All animals were fully conscious during intrathecal lidocaine injection. After optimal flexion of the lumbar spine in the prone position, each rat received a subcutaneous injection of 100 μl of 1.0% lidocaine into the lumbar 4-5 (L_4-5_) intervertebral space. Next, 50 μl of 1% lidocaine was injected into the right and left paraspinal spaces to a depth of 0.5 cm, and 0.5 cm from the mid-point of the longitudinal line of the L_4-5 _intervertebral space [19]. A 30 gauge needle attached to a 100 μl syringe (Hamilton, Reno, NV, USA) was inserted intrathecally through the midline of the L_4-5 _intervertebral space after local infiltrative anesthesia. After the needle was passed through the ligamentum flavum and the dura mater, entry into the intrathecal space was confirmed by a change in resistance and movement of the tail [19]. A 2% solution of lidocaine (50 μl, 1 mg total) was administered. Rats showing of lower limbs paralysis and without pain withdrawal response as application of Adson forceps on lateral paws were included in group P. Rats with unilateral blockades were excluded from the analysis.

### Induction of Neuropathic Pain by SNL

All surgical procedures were performed under isoflurane/O_2 _anesthesia. With the animal placed in a prone position, left paraspinal muscles were separated from spinous processes at the L_4_-S_2 _level. In Group S, the left L_6 _transverse process was removed to expose the L_4 _and L_5 _spinal nerves. In Groups L and P, the left L_5 _spinal nerve was isolated and tightly ligated with 6-0 Dexon. After complete hemostasis by light compression, the wound was sutured with 3-0 silk thread. On the second day after surgery, rats that dragged their feet during movement were excluded from the analysis.

### Behavioral Testing

#### Latency of foot withdrawal from a noxious heat stimulus

The latency of foot withdrawal from a noxious heat stimulus was measured as described by Kim and Chung [20]. Briefly, each rat was placed on a device consisting of a light box with a glass plate on top (Model 7370 Plantar Test, Ugo Basile, Varese, Italy). Rats were allowed to acclimatize to the environment. After cessation of exploratory behavior (typically 5-10 minutes), a movable radiant heat generator was placed beneath the glass floor. The glass plate was heated by a beam of light projected through a hole (2 × 5 mm) in the light box. A photocell automatically turned off the light when it sensed that the rat had lifted its foot, allowing measurement of the time between light-on and light-off. If the rat did not react after 20 seconds, the heat source was automatically switched off to avoid tissue damage. The time from application of the heat source to withdrawal of the hindpaw was defined as the foot-withdrawal latency. Five minutes were allowed between stimulations; withdrawal responses were measured five times for each hindpaw and the average response time was recorded. Spontaneous movements during heating were not recorded. To ensure that the heat stimulus remained at a constant intensity of 190 mv/m^2 ^throughout the test, the device was calibrated with a Heat-Flux I.R. Radiometer (Model 37300, Ugo Basile, Italy).

#### Latency of foot-withdrawal response to a mechanical stimulus

Rats were first acclimatized to the mechanical stimulus-testing environment, which was a metal mesh floor covered by a transparent plastic dome (8 × 8 × 18 cm). A Dynamic Plantar Aesthesiometer (Ugo Basil, Italy) filament underneath the metal mesh was applied perpendicular to the outer mid-plantar surface of the paw. The filament controller was preset to a force of 50 g and a duration of 20 seconds (increasing force from 0 to 50 g in 20 seconds). An automated test machine was used to apply a mechanical stimulus with a 2 mm diameter metal rod in increments of 2.5 g/s to either hind paw until an abrupt foot-withdrawal response was elicited, which was considered the sensitivity threshold for that paw. When rapid withdrawal of the paw was observed, the duration and force intensity were recorded with approximately 0.1 g sensitivity. Each measurement was repeated five times at intervals of approximately 3 minutes for each hind paw. The paw-withdrawal threshold was determined by averaging measurements. In each animal, hindpaws were tested separately and alternately.

### Measurement of Sodium Channels in DRGs by Western Blotting

After surgery, left L_5 _DRGs were removed from Groups L and P. The L_5 _DRG was also removed from the sham group (Group S) to provide a normal control. For each group (n = 8/group), DRGs were removed on POD_3 _(n = 4) and POD_7 _(n = 4).

Total cell proteins were homogenized and extracted using RIPA buffer (50 mM Tris pH 7.4, 150 mM NaCl, 1 mM EDTA, 0.1% SDS, 1% NP-40, 0.5% sodium deoxycholate), containing protease inhibitor cocktail (Roche, Germany). Homogenates were centrifuged at 12,000 rpm for 20 minutes at 4°C, and the supernatant protein concentration was determined using a Bio-Rad Protein Assay Kit (Bio-Rad, USA). Total protein (50 μg) was fractionated by sodium dodecyl sulfate-polyacrylamide gel electrophoresis on 8% gels and blotted onto polyvinylidene fluoride membranes (Millipore, MA, USA). Primary antibodies (rabbit anti-Nav_1.3 _and Nav_1.8_; Alomone Labs, Jerusalem, Israel) were detected with horseradish peroxidase-conjugated mouse anti-rabbit antibody (Santa Cruz, Biotechnology, Santa Cruz, CA, USA). Antibodies were detected using an enhanced chemiluminescence western blotting detection and analysis system (Amersham Biosciences, Tokyo, Japan). The density of Nav_1.3 _and Nav_1.8 _bands were measured and normalized to the density of the corresponding loading control (β-actin). The Nav_1.3 _and Nav_1.8 _band-density values for Groups L and P relative to those for Group S were calculated on POD_3 _and POD_7_.

### Tissue Preparation and Immunohistochemical Detection of Microglia and p-p38 levels

Activation of microglia in the L_5 _spinal cord segment following SNL was evaluated. Animals in Groups L, P and S (normal controls) were anesthetized with thiopentone 60 mg/kg and perfused with 0.9% saline followed by 4% paraformaldehyde in 0.1 M phosphate buffer (pH 7.4). For each group (n = 6/group), L_5 _spinal cords were removed on POD_3 _(n = 3) and POD_7 _(n = 3). Dissected tissues were then fixed in 4% paraformaldehyde and saturated in 10%-30% sucrose in 0.01 M phosphate-buffered saline (pH 7.4). After embedding the tissues in O.C.T. compound, 12 μm sections were cut with a cryostat and mounted on glass slides for immunostaining.

Microglia proliferation in the spinal cord was assessed immunohistochemically using a mouse monoclonal anti-OX-42 primary antibody (mouse anti-rat-CD11b, 1:80, Serotec, Indianapolis, USA) and an Alexa Fluor 488-conjugated goat anti-mouse IgG secondary antibody (Invitrogen, UK). Microglial activation was evaluated using anti-p-p38 (1:200; Cell Signaling, Beverly, MA, USA) as a primary antibody and Cy3-conjugated goat anti-rabbit IgG as a secondary antibody. Control experiments were performed without primary antibody and yielded only background levels of signal. The immunoreactivity of sections was examined and images were captured with an Olympus LSM_5 _fluorescence microscope. Immunofluorescence staining in the spinal cord was quantified by measuring microglia- and p-p38-positive staining areas in the ipsilateral dorsal horn of each section using a computerized image-analysis system. The background fluorescence intensity of each tissue section was also determined and subtracted from the values obtained. Fluorescence intensities exceeding this background were considered true intensities. Six sections were measured in each rat, and the average density of microglia and the intensity of p-p38 staining were determined for each group.

### Double-Immunofluorescence Labeling

For double-immunofluorescence labeling, sections were incubated for 48 hours at 4°C with a mixture of anti-p-p38 MAPK antibody (1:100) and mouse monoclonal anti-OX-42 antibody (CD11b, 1:100; Serotec, Indianapolis, USA). Cy3- or Alexa Fluor 488- conjugated secondary antibodies were used as appropriate to visualize bound primary antibodies. The stained sections were examined with a fluorescence microscope, and images were captured with a CCD spot camera.

### Histological Staining

L_5 _spinal cord specimens were collected, fixed in 10% neutral-buffered formalin for several hours and embedded in paraffin. 3-4 μm sections were cut, stained with hematoxylin and eosin (H&E) and viewed under a light microscope.

### Statistical Analysis

Differences in western blotting among groups were assessed by one-way ANOVA, followed by Scheffe's test for multiple *post hoc *analyses where appropriate. Comparisons of behavioral responses, microglia proliferation, and p-p38 activation among groups were performed using Mann-Whitney U tests. SPSS 15.0 software (SPSS, NY, USA) was used for all statistical analyses, and a *P*-value < 0.05 was considered statistically significant. Specific significance levels are indicated in figure legends.

## Results

None of the animals exhibited autotomy, body-weight loss, ventroflexion, or dragging of the hind paw during forward movement. In Group S, immunohistochemical staining results from ipsi- and contralateral sides were statistically indistinguishable, and there were no significant differences between left and right hind paws in behavioral tests. Abnormal histopathological responses to intrathecal lidocaine, including obvious demyelination of spinal root or inflammatory cell infiltration into the spinal cord, were absent in all rats.

### Behavioral testing

**Figure 1:**
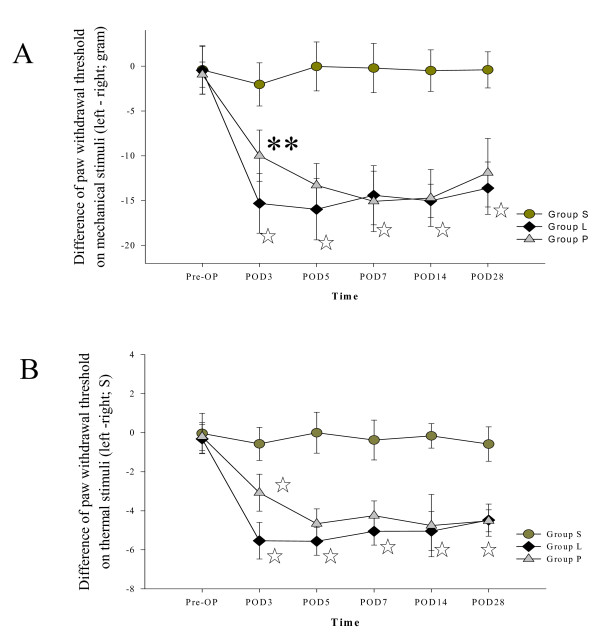
**Intrathecal lidocaine pretreatment attenuated neurobehavioral responses to thermal and mechanical stimuli**. (A) Mechanical responses. Comparison of mean paw withdrawal in response to a mechanical stimulus (F = 50 g; T = 20 seconds) between ipsilateral (left) and contralateral (right) sides in each group. Differences in mean paw withdrawal thresholds between hindpaws were significantly higher in Group L than in Group P on POD_3 _(**P <0.01). (B) Hyperalgesic responses. On POD_3_, the difference in mean withdrawal threshold between left and right hindpaws was significantly larger (☆*P <*0.001) in Group L than in Group P. Mean withdrawal thresholds did not significantly differ between left and right hindpaws in Group S. Data represent means ± SE.

Figure 1A shows differences in withdrawal threshold to a mechanical stimulus between left and right paws. The average baseline withdrawal threshold forces for left/right paw were 33/33.4 g in Group S, 34.9/35.6 g in Group L and 34.3/35.1 g in Group P. Throughout the study, all measurements of right paw withdrawal remained in the 28-36 g range. The difference in force-induced withdrawal thresholds between hindpaws (mechanical allodynia) increased significantly in Group L; intrathecal lidocaine pretreatment shortened the difference during the first 3 days (*P *< 0.01, Group L vs. P). Figure 1B shows the differences in withdrawal threshold to thermal stimuli between left and right paws. The average baseline thermal withdrawal thresholds for left/right hindpaws were 11.0/11.2 seconds in Group S, 10.8/11.0 seconds in Group L, and 10.9/10.8 seconds in Group P. In Group L, withdrawal latency of the left hindpaw was shortened after SNL; intrathecal lidocaine pretreatment blunted the responses on POD_3 _(*P *< 0.001, Group L vs. P).

### SNL modulated Nav_1.3 _and Nav_1.8 _expression in injured DRGs

**Figure 2:**
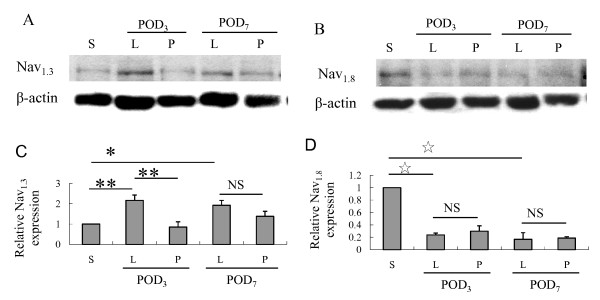
**Western blot showing that intrathecal lidocaine pretreatment decreased Nav_1.3 _up-regulation in injured DRGs after SNL **(A). Quantification of Nav_1.3 _band densities showed Nav_1.3 _expression was up-regulated after SNL, but was decreased on POD_3 _in Group P (C). Western blot and quantification of Nav_1.8 _band densities showing that intrathecal lidocaine pretreatment did not reduce Nav_1.8 _down-regulation in Group P (B, D). (*p < 0.05, **p < 0.01,  ☆*P <*0.001, NS: not statistically significant; ANOVA with post hoc Scheffe's test.)

SNL dysregulated DRG sodium channel expression in injured DRGs, up-regulating Nav_1.3 _and down-regulating Nav_1.8_. Compared with the ligated-only group, intrathecal lidocaine pretreatment reduced Nav_1.3 _up-regulation on POD_3 _(*p *= 0.003) but not on POD_7 _(*p *= 0.36; Figure 2). However, intrathecal lidocaine pretreatment had no significant effect on Nav_1.8 _down-regulation.

### Increased proliferation and activation of microglia in the spinal cord after SNL

**Figure 3:**
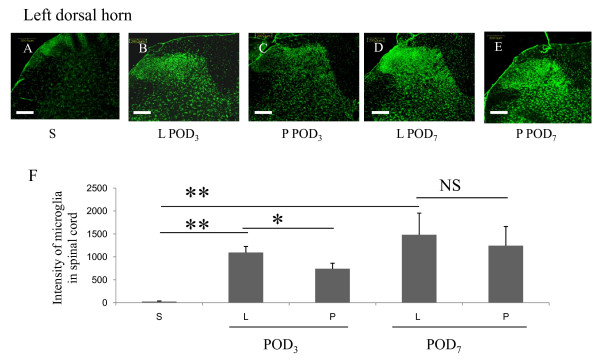
**Intrathecal lidocaine pretreatment suppressed microglia-specific immunoreactivity induced by SNL in the spinal cord**. Group S showed weak immunoreactivity of the microglial marker OX-42 in the left dorsal horn (A). In Group L, SNL induced a significant increase in microglia immunoreactivity in the ipsilateral spinal cord on POD_3 _(B) and POD_7 _(D). Intrathecal lidocaine pretreatment (Group P) decreased microglia immunoreactivity on POD_3 _(C) but not on POD_7 _(E). Data represent means ± SE (*p < 0.05, **p < 0.01, NS: not statistically significant; Mann-Whitney U test). Scale bars, 200 μm.

**Figure 4:**
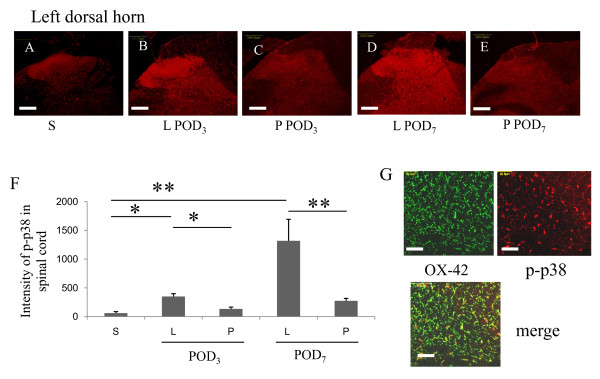
**Intrathecal lidocaine pretreatment moderated the increase in p-p38 immunoreactivity induced by SNL**. Group S exhibited weak p-p38 immunoreactivity in the left dorsal horn (A). In Group L, p-p38 immunoreactivity was significantly increased in the left dorsal horn on POD_3 _(B) and on POD_7 _(D). Intrathecal lidocaine pretreatment (Group P) decreased SNL-induced p-p38 immunoreactivity on POD_3 _(C) and POD_7 _(E). Quantification of p-p38 immunoreactivity revealed significant increases on POD_3 _and POD_7 _after SNL. Intrathecal lidocaine pretreatment reduced p-p38 immunoreactivity after SNL (F). Merged images from OX-42 (green) and p-p38 (red) in the ipsilateral L_5 _spinal cord on POD_3 _showed p-p38 colocalization with OX-42 (G). Data represent means ± SE (*p < 0.05, **p < 0.01; Mann Whitney U test). Scale bars, 200 μm (A-E) and 50 μm (G).

SNL induced an increase the activation status of residual microglia in the spinal cord, as evidenced by increased OX-42 expression and increased p-p38 immunoreactivity. In Group S, low levels of OX-42 immunoreactivity in the spinal cord were observed (Figure 3A). Multiple activated microglia were observed in the ipsilateral dorsal horn of the spinal cord after SNL, as shown in Figure 3B (POD_3_; *p *= 0.004) and 3D (POD_7_; *p *= 0.008). Many activated microglia in Group L were p-p38 immunopositive after nerve injury. Compared with Group S, which showed low basal levels of p-p38, Group L exhibited a higher intensity of p-p38 staining in the L_5 _ipsilateral dorsal horn on POD_3 _(Figure 4B; *p *= 0.034, Group S vs. L), which was further increased on POD_7 _(Figure 4D; *p *= 0.005, Group S vs. L). Double-immunofluorescence labeling with OX-42 (for microglia) and p-p38 (for activated microglia) revealed colocalization of p-p38 and OX-42 (i.e., dual-immunopositive cells) in the spinal cord (Figure 4G).

### Intrathecal lidocaine pretreatment reduced microglial proliferation and activation (p-p38 levels) in the spinal cord

Compared with ligation only, intrathecal lidocaine pretreatment decreased the intensity of OX-42 immunoreactivity in the left spinal dorsal horn on POD_3 _(Figure 3C; *p *= 0.026), but not on POD_7 _(Figure 3E; *p *= 0.289). The levels of p-p38 immunoreactivity were significantly lower on both POD_3 _(Figure 4C; *p *= 0.034) and POD_7 _(Figure 4E; *p *= 0.002). Taken together, these results indicate that intrathecal lidocaine pretreatment before SNL reduced activation of spinal microglia, as evidenced by decreased OX-42 immunoreactivity and decreased p-p38 levels.

## Discussion

A single intrathecal injection of lidocaine, a standard regional anesthetic procedure, is routinely performed in lower limb surgery patients. It provides a stable hemodynamic state during surgery, promotes smooth recovery and attenuates immediate postoperative pain. However, there is no consensus on the use of intrathecal lidocaine pretreatment for patients with nerve damage who require surgery.

The tetrodotoxin-sensitive sodium channel, Nav_1.3_, is expressed only during early stages of development and is difficult to detect in the nervous system of adult rats. After peripheral axotomy, Nav_1.3 _expression in injured DRG neurons may increase within 8 hours, resulting in a reduced action potential threshold and/or a relatively high firing frequency [21]. In this study, intrathecal lidocaine pretreatment inhibited Nav_1.3 _expression in injured DRGs on POD_3_. This result is consistent with studies of pain-related behavior, which have shown a positive correlation between increased Nav_1.3 _expression in injured DRGs and peripheral nerve axotomy. Increased Nav_1.3 _expression in axotomized DRG neurons shifts the threshold to a more hyperpolarized overshooting action potential that requires less depolarization for activation and boosts ramp currents [22]; the rate of recovery from inactivation is also accelerated in injured neurons [23]. However, intrathecal administration of Nav_1.3 _specific antisense oligonucleotides, which reduces DRG immunoreactivity by 50%, does not attenuate mechanical or cold allodynia [24]. Moreover, neuropathic pain behavior is unaffected in global and conditional Nav_1.3 _knockout mice [25]. These reports indicate that increased Nav_1.3 _expression does not substantially affect the behavioral hypersensitivity of injured nerves. Further study is needed to demonstrate whether Nav_1.3 _plays a substantial role during neuropathic pain.

Peripheral nerve injury induces profound changes in microglia, including hypertrophy, proliferation and up-regulation of cell surface markers; it also modulates pain signals in the dorsal horn and induces neuropathic pain [26]. Blocking microglial activity and inhibiting microglial proliferation are both effective in preventing or delaying neuropathic pain [27,28]. Although the exact roles of activated spinal microglia and neurons in pain processing are still undetermined, an increase in p-p38 levels in activated microglia after peripheral nerve injury is reported to be a major cause of hypersensitivity to pain [28-30]. Intrathecal administration of lidocaine after peripheral nerve injury is known to reverse well-developed tactile allodynia [14]. A rat model of chronic constriction injury also indicated that lidocaine reverses tactile allodynia by attenuating phosphorylation of p38 in spinal cord microglia [12]. In the current study, rats pretreated with intrathecal lidocaine exhibited decreased spinal microglia activation on POD_3 _and diminished p-p38 levels on POD_3 _and POD_7 _after SNL. This indicates that the pretreatment reduces neuropathic pain not only by inhibiting the proliferation of spinal microglia, but also by reducing spinal microglial activity through a decrease in p38 phosphorylation.

Although the Nav_1.8 _has important roles in both normal pain function and inflammatory pain, in this study, Nav_1.8 _expression in injured DRGs was reduced after nerve axotomy. Rat models of neuropathic pain associated with axotomy reveal a substantial reduction in Nav_1.8 _mRNA and protein levels in injured DRG neurons [31,32]. However, specific knockdown of Nav_1.8 _by intrathecal administration of an antisense oligodeoxynucleotide is reported to exert anti-allodynic and anti-hyperalgesic actions following SNL [33]. A potent and highly selective Nav_1.8 _channel inhibitor has been shown to block spontaneous and evoked action potentials in injured DRG neurons [34]. Therefore, measuring expression of Navs in the vicinity of injured DRGs could be an effective method for assessing neuropathic pain.

In the present study, lidocaine (2 mg) was locally applied to L_4-5 _subcutaneous tissues and vertebral paraspinal muscles to provide pain relief and to prevent rats from moving during lumbar intrathecal lidocaine injection. But the ability of systemically absorbed lidocaine to attenuate neuropathic pain following local injection is a matter of debate. In rats, lidocaine administered intravenously at doses of 1, 4 or 10 mg/kg did not abolish or diminish brain activity in response to innocuous or noxious stimulation. In fact, intravenous doses of 4 and 10 mg/kg lidocaine have been shown to enhance sensory cortex responses to acute nociceptive stimulation, increasing the activated cortical volume by 50%-60% [35]. A large lidocaine intravenous dose (10 mg/kg bolus in 3 minutes, followed by 180 μg/kg/min for 1 hour) provides relief from neuropathic pain induced by chronic constriction of the sciatic nerve [36]. Furthermore, local application of lidocaine to incisional skin and exposed spinal nerves has been demonstrated to attenuate postoperative peri-incisional skin allodynia [37]. But the site of local lidocaine infiltration in the present study was away from incised skin and no lidocaine was applied to spinal nerves. Therefore, the dose of local lidocaine injected into L_4-5 _subcutaneous tissue and infiltrated into paraspinal muscles should not ameliorate L_5 _SNL-induced allodynia and hyperalgesia.

## Conclusions

Peripheral nerve injury caused by trauma or surgery may up-regulate Nav_1.3 _and down-regulate Nav_1.8 _in injured DRGs, and activate spinal microglia, as evidenced by increased levels of phosphorylated p38 in the spinal cord. A consequence of this dynamic is the development of neuropathic pain. Intrathecal lidocaine pretreatment alleviates neuropathic pain by modulating Nav_1.3 _expression and decreasing spinal microglial activation. Although the effect of intrathecal lidocaine pretreatment in this animal model of spinal nerve injury was limited to 3 days, this treatment has excellent potential for treating immediate neuropathic pain.

## Competing interests

The authors declare that they have no competing interests.

## Authors' contributions

KIC is the primary author for this manuscript. He conceived, organized and implemented this research. He interpreted the data, guided intellectual discussions of the results and drafted and revised the manuscript. CSL served as supervisor and assisted in the design of the study. FYW, HCW and STH are responsible for the integrity of the data and the accuracy of the data analysis. LLC participated in the study design, coordinated the study, performed the statistical analyses, and drafted and revised the manuscript. HPT executed behavioral examinations in the study. ALK supervised the research program and contributed to integrate the research team. All authors have read and approved the final manuscript.

## Pre-publication history

The pre-publication history for this paper can be accessed here:

http://www.biomedcentral.com/1471-2377/11/71/prepub
